# Association of anterior thalamic radiations alterations with impulsivity in patients with borderline personality disorder

**DOI:** 10.1192/j.eurpsy.2025.551

**Published:** 2025-08-26

**Authors:** L. Franczak, P. Podwalski, B. Dawidowski, J. Samochowiec

**Affiliations:** 1 Department of Psychiatry, Pomeranian Medical University, Szczecin, Poland

## Abstract

**Introduction:**

The anterior thalamic radiations refers to fiber pathways that connect the anterior nuclear group of the thalamus and the midline nuclear group of the thalamus with the frontal cortex – a region linked to complex cognitive processes such as impulse control and decision making abilities. Typically characterized by risky behaviors without prior consideration or planning impulsivity is a hallmark feature of Borderline Personality Disorder (BPD). Structural alterations in the anterior thalamic radiations may disrupt cognitive functions and potentially exacerbate impulsivity, in individuals diagnosed with BPD.

**Objectives:**

The objective of this research is to evaluate whether individuals with borderline personality disorder (BPD) exhibit abnormalities in the anterior thalamic radiations (ATR) compared to healthy controls (HC) and their potential association with impulsivity.

**Methods:**

This research conducted an analysis using diffusion tensor imaging (DTI) being conducted on 90 individuals (aged between 18 and 43), fifty five individuals diagnosed with borderline personality disorder compared to forty five healthy individuals. The study utilized fractional anisotropy (FA ) to investigate the integrity of the thalamic radiation (ATR). Impulsivity was measured through Eysenck’s Impulsivity Inventory questionnaire. The study examined differences in FA values among the two groups. Looked into the potential correlation, between ATR integrity and levels of impulsivity.

**Results:**

The BPD group had statistically significantly higher scores on the impulsivity subscale than the HC group (U = 1672.5, p < 0.001), which persisted after correction for multiple comparisons (p < 0.001). No statistically significant between-group differences were found on the venturesomeness (U = 889, p = 0.513) and empathy (U = 1097.5, p = 0.276) subscales. No statistically significant differences between groups were observed in the right ATR (U = 849, p = 0.326). Only a trend for higher FA values in the left ATR in HC was observed (U = 760, p = 0.084). After correction for multiple comparisons, the trend disappeared (p = 0.168). There were no statistically significant correlations of mean FA with IVE subscales scores, regardless of stratification.

**Conclusions:**

Despite past research suggesting a link between ATR alterations and impulsivity, this study found no such correlation in individuals with BPD. This highlights the need for further research, using imaging methods and larger samples, to better understand the neurological basis of impulsivity in BPD.

**Disclosure of Interest:**

None Declared

